# Supplementary material for: Pay-it-forward influenza vaccination among older adults and children: A cost-effectiveness analysis in China
Source: PLOS Glob Public Health. 2023 Aug 31;3(8):e0001590. doi: 10.1371/journal.pgph.0001590 (PMC10470923; doi:10.1371/journal.pgph.0001590)
Supplement: S1 Appendix — (DOCX) [file pgph.0001590.s001.docx]

*S1 Appendix: English version of questionnaire administered to study participants*

Influenza vaccination for children and elderly people

About this study:

You are invited to participate in this influenza vaccination study. The results of this study can provide evidence for the government to develop better intervention strategies to promote influenza vaccination in China and prevent seasonal influenza among at-risk populations.

Details of this study:

You will need to fill out a questionnaire. This questionnaire will ask about your socio-demographic information and opinions towards influenza vaccine. To protect your privacy, all your answers will be transformed into digital formats and password encrypted. If you are eligible to participate in this program, out project personnel will provide you with more detailed information. To confirm whether you are qualified to participate in this program, we will first ask you a few screening questions. If you are not eligible for this program, we will not keep your survey information.

Note: If the participant is a child, the guardian will need to consent on the child’s behalf and fill in the questionnaire. For the purpose of equality, only one quota is provided for each participating family, either for a child or an elderly individual.

1.Which category are you belong to____

○Caregivers of a 3-8 year-old children

○Elderly people (>=60)

2. Your age (fill in a number) ____

3. Your gender（male-1，female-2）___

4. Before today's vaccination, did you or your family participate in a research program called ‘Pay-it-forward’ (influenza vaccination)? [Pay-it-forward version]

○Yes (Please skip to the end of the questionnaire and submit the answer sheet)

○No

5. Have all of your children and elderly people in your family been vaccinated against influenza in the past year?

○Yes (Please skip to the end of the questionnaire and submit the answer sheet)

○No

A. Basic information

6. Ethnicity

○Han

○Other, please specify： ____________

7. Your highest education level:

○Primary school

○Junior middle school

○High school

○Undergraduate or college

○Postgraduate or above

8. Your occupation is:

○Student

○Civil servant

○Farmer

○Ordinary workers (blue collar)

○Company staff (white collar)

○Technical personnel

○Unemployed or retired

○Other：___________________*

9. Your personal monthly income:

○0-1000 RMB/Month

○1000-5000 RMB/Month

○5000-10,000 RMB/Month

○10,000 RMB/Month or above

10. Your current legal marriage status is:

○Unmarried

○Engaged or married

○Separated or divorced

○Widowed (skip questions 6-9)

11. Age of your partner：____

12. The highest education level of your partner:

○Primary school

○Junior middle school

○High school

○Undergraduate or college

○Postgraduate

13. The occupation of your partner is:

○Student

○Civil servant

○Farmer

○Ordinary workers (blue collar)

○Company staff (white collar)

○Technical personnel

○Unemployed or retired

○Other：___________________*

14. Your partner’s personal monthly income

○0-1000 RMB/Month

○1000-5000 RMB/Month

○5000-10,000 RMB/Month

○10,000 RMB/Month or above

15 Do you have children in your family?

○Yes

○No (skip question16)

16. How many children are there in your family? (fill in your children's age and sex in chronological order, for example, Child 1, 2 years old, male-1 or female-2)

○Child 1

Age_____

Sex_____

○Child 2

Age_____

Sex_____

17. Do you have elderly people in your family?

○Yes

○No

18. How many elderly people (>=60 years) are there in your family? Age and gender?

○Elderly individual 1

Age:

Sex:

○Elderly individual 2

Age:

Sex:

B. Participation in “Pay-it-forward” / “Standard-of-care” / Free vaccination programme

19. Main purpose of coming to the clinic？

○See a doctor for myself

○Take my child(ren) to see a doctor

○Take my child(ren) to vaccinate

○Accompany elderly family members to see a doctor

○Other： ____________________ *

20. [Standard-of-care version] The researcher has introduced the influenza vaccine / [Free vaccination version] and free vaccination programme / [Pay-it-forward version] and ‘Pay-it-forward’ program to you. Would you like to participate in the program to vaccinate your child (or yourself if older than 60 years) today?

○Yes

○No（Please skip to the question 27）

21. Who would you like to vaccinate today?

○My Child

○Elderly family member

22.The information of the vaccination recipient?

○Sex___

○Age___

23. Are you willing to donate some money to the next family to get the same influenza vaccination? [Pay-it-forward version]

○Yes

○No（Skip to the Question 25）

24. How much would you like to donate to the next family? [Pay-it-forward version]

○200 RMB (support one family to get vaccination (1elderly+1children))

○150 RMB (support 1 elderly or 3 children get vaccination)

○100 RMB (support 2 children get vaccination)

○50 RMB (support 1 children to get vaccination)

○Other amount_____

25. If you want your family to vaccinate, what is the main reason?

○Children in my family are easy to get seasonable flu

○Elderly people in my family are easy to get seasonable flu

○Both my children and the old people in my family are easy to get seasonable flu

○I’m easy to get seasonable flu

○Recommended by my friends/family member

○Recommendation by medical personnel at the clinic (irrelevant to ‘Pay it forward’ program)

○The ‘Pay-it-forward’ program [Pay-it-forward version]

○The free vaccination program [Free vaccination version]

○Other, please note： ______________ *

26. What do you think are the benefits of the ‘Pay it forward’ program? [Pay-it-forward version]

[Multiple choice]

□Other families’ donation can lower my financial burden

□I learn about influenza vaccines that can prevent flus

□Can promote more families to be vaccinated against influenza

□Can reduce the spread of influenza

□Spread love and warmth within the community

□Other： _______________________ *

27. If you don't want to vaccinate your child or an elderly individual in your family today, why not? [multiple choices] * (if you answered "yes" to question 2, skip this item)

□I don't know enough about seasonal influenza

□I'm not sure about the effect of the influenza vaccine

□My family don't need vaccinations

□It’s too cumbersome

□I'm worried about the side effects

□Other, please specify: _______________*

C. Vaccine related information

28. Have you heard about the flu vaccine before this program?

○Yes ○No

29. In general, I think influenza vaccine is important. [Single choice] *

○Strong disagree ○Disagree ○Agree ○Strongly agree

30. In general, I think the influenza vaccine is safe. [Single choice] *

○Strong disagree ○Disagree ○Agree ○Strongly agree

31. In general, I think the influenza vaccine is effective. [Single choice] *

○Strong disagree ○Disagree ○Agree ○Strongly agree

32. Has your child ever been vaccinated against influenza?

(if there is no child in your family, please skip this question) [Single choice] *

○Yes ○No

33. Have elderly people in your family ever been vaccinated against influenza

(if there are no elderly individuals in your family, please skip this question) [Single choice] *

○Yes ○No

34. Have you ever been hesitant about having your child or elderly family members to get the flu vaccine (except for allergies)? [Single choice] *

○Yes ○No

35. Have you ever postponed your child or elderly family members to get the flu vaccine (except for allergies)? [Single choice] *

○Yes ○No

36. Have you ever been refused to have your child or elderly family members to get the flu vaccine (except for allergies)? [Single choice] *

○Yes ○No

37. Have you ever heard about negative information about influenza vaccine?

○Yes ○No

38. Do any of your friends or relatives object to the influenza vaccination?

○Yes ○No

39. Have you or people around you had adverse reactions to influenza vaccination?

○Yes ○No

40. Do you trust the advice provided by the medical personnel in the clinic on influenza vaccine?

○Yes ○No

41. Is price of the vaccine a barrier for your child and/or elderly individuals in your family to get the influenza vaccine?

○Yes ○No
