# Supplementary material for: Pay-it-forward influenza vaccination among older adults and children: A cost-effectiveness analysis in China
Source: PLOS Glob Public Health. 2023 Aug 31;3(8):e0001590. doi: 10.1371/journal.pgph.0001590 (PMC10470923; doi:10.1371/journal.pgph.0001590)
Supplement: S2 Appendix — (DOCX) [file pgph.0001590.s002.docx]

*S2 Appendix: Flow and organisation of pay-it-forward programme implementation in this study*

1. A preparatory workshop was held beforehand to brief and train nurses and clinic coordinators in the three study sites regarding the delivery of the seasonal influenza vaccination programme.
2. Costs of pay-it-forward-specific training and items such as the cost of postcard design by research assistants and the initial conception of the pay-it-forward programme during the preparatory briefing were annualised over a period of three years at a discount rate of 0.03 as it was expected that these resources and skills could be passed on (e.g. the postcard design could be reused in future iterations of the programme).
3. Fixed costs mainly consisted of the cost of hiring doctors as vaccinators and quality checkers for the vaccination programme, who would be working in the clinic regardless of the recruitment of participants for vaccination (i.e. vaccine uptake).
4. Recurrent costs included the additional recruitment time of nurses during the initial consultation when screening for the eligibility of receiving seasonal influenza vaccination. More time was needed for pay-it-forward arm because the recruiting nurse would have to explain the programme and convince the patient to donate to the pool.
