# Supplementary material for: Pay-it-forward influenza vaccination among older adults and children: A cost-effectiveness analysis in China
Source: PLOS Glob Public Health. 2023 Aug 31;3(8):e0001590. doi: 10.1371/journal.pgph.0001590 (PMC10470923; doi:10.1371/journal.pgph.0001590)
Supplement: Attachment — Submitted filename: Rebuttal Letter to the Reviewers.docx [file pgph.0001590.s005.docx]

Dear Editors of PLOS Global Public Health,

We thank the reviewers for taking the time to provide comprehensive feedback on the manuscript. The revisions the reviewers have suggested have been addressed in this new iteration of the paper.

The following are the team’s responses to the reviewer’s feedback:

a. We have removed corrected the grammatical error pointed out by the reviewer. The sentence now reads “We conducted a three-arm quasi-experimental study to assess the effectiveness of a pay-it-forward strategy to increase influenza vaccine uptake in China compared to standard-of-care and free vaccination strategies”
b. Only clinics that provided primary care services were eligible to participate. Additionally, participating clinics had a appropriate stock of influenza vaccines and trained healthcare providers. Information on research participant eligibility was also added to the manuscript. In the methods section, we have included this information on inclusion criteria for clinics to be involved in the study.
c. Without taking the factor of the amount of donations into consideration, we may look at the deterministic sensitivity analysis (DSA) for more information. The DSA attempts to reflect the best and worst-case scenario. In the worst-case scenario, when vaccine uptake in the PIF arm drops to 50% and the SOC arm remains at 37%, the ICER increases to 66.31 USD per person vaccinated.

Depending on the cost-effectiveness threshold that the local government is willing to pay, this may yet still be cost-effective. For reference, a systematic review by Munk et al, 2019 published in BMC Health Services Research which evaluated the cost-effectiveness of interventions to improve infant immunization coverage in low- and middle-income countries reported ICERS ranging from $0.66 to $161.95 per child vaccination. Taking the average of the two figures, the worst-case scenario in the DSA at 66 USD is still well below 80 USD per vaccination.

However, an important factor to note would be the amount of donations, as stated in the DSA. As the vaccine uptake in the pay-it-forward arms indirectly affects the donation amount received (i.e. more vaccinations = more donations = potentially higher amount of donations reimbursing cost), naturally a higher uptake would lead to a higher chance of the PIF arm being cost-effective. However, due to the variable nature of the donation amounts, it is hard to predict whether each iteration of the PIF programme will generate the same amount of donations. Thus, it is difficult to pinpoint exactly at what minimum vaccine uptake threshold it is required to keep the WTP threshold at a certain level.

d. We investigated the efficacy of the pay-it-forward intervention at the community evel, thus the health policy implications of the study are also relevant to community level stakeholders. We recognize that in order to implement a pay-it-forward program, more information on the health effects and broader economic consequences is needed. In the limitations section of the manuscript, we state the following: “In order to effectively implement pay- it-forward operates, more must be known about the mechanisms of why pay-it-forward works through implementation and mixed methods research.”

Low average donation amounts in pay-it-forward programs requires sufficient health information dissemination strategies from public health practitioners. Further clarification on barriers to keeping cost low for health policy interventions was added in the discussion section.

e. Inter-site differences in cost-effectiveness across the three study sites were not assessed. However, statistical analysis of the population demographics of the donors by study site had been performed.

When looking at the proportion of donations contributed by each study site, the rural site contributed 30% (178 USD), the suburban site contributed 44% (256 USD), and the urban site contributed 26% (151 USD) of total donations (585 USD).

There was a statistically significant difference in the proportion of people who donated in the urban site at 50% (25/50) compared to the rural and suburban sites at 80% (40/50) and 84% (42/50) respectively. However, the donation amount per person in the urban area was higher, so the three sites contributed about equally to the total amount of donations as detailed above.

The vaccine uptake across the three sites in each arm was also similar. For the urban site, even though the site had the lowest PIF arm vaccine uptake, the donations were on average higher in amount, with around 60% being more than 50 RMB. With such variabilities in the donation amounts, it is not likely that there would be significant inter-site differences in cost-effectiveness across the three study sites or across study arms. We have added this sentence to the limitations section: “Our study was not designed to assess the inter-site differences in cost-effectiveness. However, the total amount donated and the vaccine uptake was similar across sites.”

Best Regards,

Priya Kosana
